# The concordance game: A simple tool to estimate breath hold swimming performance and to teach dynamic apnea

**DOI:** 10.3389/fpsyg.2022.1046533

**Published:** 2022-11-21

**Authors:** Damien Hello, Damien Vitiello, Luc Collard

**Affiliations:** Université Paris Cité, Institut des Sciences du Sport-Santé de Paris, Paris, France

**Keywords:** Physical Education, dynamic apnea, self-knowledge, concordance, performance, game theory, teaching games, motor praxeology

## Abstract

**Introduction:**

Swimming is composed of several phases. One of them is done underwater in apnea. Although this phase takes an important part of the performance, it is not taught much because of the risk it entails. At the same time, learning apnea can reduce the fear of immersion and, thus, reduce the number of drownings. The pedagogy used in this paper comes from game theory. This paper tested an apnea game based on the agreement between self-prediction and realization of the task.

**Methods:**

Considering the preliminary level of the 33 sports students involved, the game offered two choices: swimming apnea over 15 or 20 m with a distribution of payoffs depending on the actual achievement (15 m estimated and less than 20 m performed = + 3 points; 15 m estimated and at least 20 m realized = + 1 points; 20 m predicted and less than 20 m realized = + 2 points; 20 m estimated and at least 20 m realized = + 4 points).

**Results and discussion:**

Concordance was favored over discordance, including in the swimmer’s comfort zone (15 m). Throughout six apneas the results showed that the structure of this game supports the improvement of the estimation of the distances swum. The “Concordance Game” could be offered in Physical Education or in a sports club to learn to swim a longer distance below the surface without forcing.

## Introduction

Human fascination and fear of water and apnea occur in every period of history and civilization. There is a major contradiction between the extraordinary human adaptations to immersion—bradycardia, decreased systolic ejection volume, peripheral vasoconstriction, splenic contraction in deep diving (Mayol, 1986)—and the terror associated with drowning, whether at school, in a swimming club or any other aquatic activity (Collard, 2018). It crosses more globally in Western history from the Middle Ages to the present (Chauvaud, 2007). This terror probably comes from the fact that underwater, even in a lighted and heated swimming pool, the external senses that usually ensure our safety are troubled. The view is blurry (Luria and Kinney, 1970), the field of vision is reduced, and sound travels five times faster while giving muffled signals (Kinney et al., 1970). The proprioceptive markers are also distorted (Counil et al., 2012; Ma, 2021). The kinesthetic sense is damaged. To be convinced, all you have to do is kneel at the bottom of a swimming pool (by draining enough air from your lungs) and try to put your arms horizontally with your eyes closed. By reopening them to control the horizontality of the arms, we might find that they are oblique—the pressure of the water (more than 800 times denser than air) distorts our sensations of movement. American swimmer, Mark Spitz, reported he was convinced that he was swimming with his arms outstretched before NASA’s pool cameras revealed that he was bending them during every underwater action (Counsilman, 1968). The labyrinthine sense is also disturbed. The human inner ear sends constant commands to the cerebellum to regain the surface (Silbernagl and Despopoulos, 2007), while evolution has endowed marine mammals with tiny semicircular canals to avoid “seasickness” in the submerged position (Thewissen, 2014). The change in the environment has disturbed our senses. It is, therefore, important to adapt to the changed proprioception and to learn new skills (Fitts, 1964).

As explained (Parlebas, 2008) in the lexicon of motor praxeology, game theory is a mathematical discipline that studies situations of confrontation or conflict from the perspective of rational decision-making. It is a theory according to which many situations are studied in order to define so-called optimal success strategies. This paper proposes to emphasize the motor action rather than the success strategy through the sporting game theory. Indeed, the aim here is to use sporting game theory as a pedagogical tool. Nobody clearly knows how to swim properly, and even fewer are able to perform an apnea for any length of time or depth, as the following examples show. When teaching swimming at school or in clubs, it is sometimes interesting to confront swimmers with immersion phases. However, from a safety point of view, this is quite challenging. In our research, we have observed an improvement in performance in dynamic apnea in students confronted with game theory, while respecting the safety rules of underwater activity.

During a pre-experimental phase realized at our university, 18 sports students in STAPS (Sciences and Techniques of Physical and Sports Activities) were estimated and then performed their maximum distance in dynamic apnea. With the exception of a few swimmers who exceeded 20 m, most swimmers returned to the surface at around 15 m. The oxygen saturation rate (O_2_)—measured 30 s before and 30 s after their apnea—reveals that the subjects only used up to 5% (SD = 2.3) of their O_2_ reserve during the dynamic apnea test (compared to 40% for a confirmed free diver measured with the same oximeter). Most students underestimate, therefore, their capacity to perform apnea.

The literature review identifying the estimation of swimming skills shows that young men are more optimistic in their estimations than young women (Howland et al., 1996; Gilchrist et al., 2000; Gulliver and Begg, 2005; Moran, 2006; MacCool et al., 2008). But these estimations are rarely verified in action or they are verified but indirectly. The difficulty is to measure aquatic performance without endangering participants (Fisher, 1981; Rudisill et al., 1993; Wiesner and Rejman, 2014; Martínez-Santos et al., 2020). In aquatic environments, knowledge of the potential gap between estimation and result of performances largely improves successful active pedagogy (Raudsepp and Liblik, 2002; Sollerhed et al., 2008; Blitvich et al., 2011; Stillwell, 2011; Wang et al., 2013). Additionally, protects from the risks of drowning (Rahman et al., 2009) and lowers the estimation of danger (Baker et al., 1993).

Most of the tests have been designed to measure skills in sports environments (over aquatic activities) and organized as a game for a player, referred to in a game theory as “play against nature.” These are stress tests and physical quality tests correlated to psychological and social variables (Sollerhed et al., 2008) or anthropometric data (Szczesny, 1983). These tests are also duels or strictly competitive play (where everything that one gains, and the other loses (Shubik, 2006; Parlebas, 2010). Based on performance through conflict, they are predisposed to risky behavior (Bay-Hinitz et al., 1994; Collard, 1998).

An analysis, proposed by Moran et al. (2012) *via* a role-playing game called “play against nature,” reveals a significant correlation between estimating and performing according to a surface swimming protocol. In the experiment, participants were asked if they were able to achieve different swimming tasks. It was a positive correlation for the following tasks: swim the farthest in 15 min, stay as long as possible on the surface while holding their breath, and swim 100 m in backstroke. They were also asked if they were able to swim underwater. Sixty-two percent of the participants answered that they could. Only 37% were really able to swim more than 20 m. The authors concluded that there is no correlation between the estimation of the performance and the reality of swimming 20 m in dynamic apnea (*r* = −0.134, Spearman’s rank correlation).

Another study (Collard et al., 2015) proposes a pure coordination game (Crawford and Haller, 1990) in which swimmers had to agree on the distance that they would swim underwater after diving into a swimming pool. The swimmers were not able to communicate with each other. To win the game, the swimmer had to surface at the same point as his partner. The results revealed that swimmers underestimate their competence when they are not subjected to a competitive challenge. In fact, they estimated their performances according to the Schelling point.

According to previous studies about game theory, strictly competitive games were sidelined (due to excessive risk-taking) as were games of pure coordination (by the lack of confrontation). The game of dynamic apnea (called “The Concordance Game” or CGame) using new skill experiences helps the external and proprioceptive stimuli to refine a body scheme (Ono and O’Reilly, 1971; Kerr, 1973; Parlebas, 1999; Berthoz, 2000).

The purpose of this paper is to improve dynamic apneas through game situations; improve self-awareness by confronting estimation of the degree of achievement (self-fulfilling prophecy) and the realization of actions (i.e., by swimming); and ensure the safety of swimmers by avoiding the risky maximal performance. Finally, this study aim at proposing a new tool that may be reinvested for swimming ability in sports training or Physical Education (Pelayo et al., 1999).

## Materials and methods

### Participants

Prior to the test, all subjects signed a written informed consent in accordance with the principles outlined in the Declaration of Helsinki in 1975. Participants were all sports science students at the school of sport sciences (i.e, Unité de Formation à la Recherche (UFR) STAPS) from the Université Paris Cité, France. Students were volunteers to participate in the CGame. The proposed swimming situations are part of their training cycle. Therefore, in case of an accident, students are insured by their university. An initial group of 53 male participants (18–20 years of age: *x* = 18.6; SD = 0.79), with the capability to swim at least 440 m in 10 min participated in this experiment. Thirty-three of the 53 participants are able to swim between 15 and 17.5 m underwater and are, therefore, selected to participate in the CGame (Table 1). The other 20 swimmers were not chosen because they had not reached the 15-m threshold or had already exceeded 20 m. None of the 33 swimmers recruited for the experiment had ever practiced apnea, either at school or in a sports club.

**TABLE 1:** Distribution of students according to the distance swum.

Distance (m)	5	7.5	10	12.5	15	17.5	20	22.5	25	>25
Number of students	0	0	0	9	**18**	**15**	9	1	0	1

Only the 33 sports students highlighted are selected for the CGame.

### Dynamic apnea game

To improve the apprehension of aquatic immersion by participants, the Concordance Game was proposed. The game was carried out in a swimming pool. The pool was 50-m long and 1.8-m deep. Moreover, there were no markers that could give information about the distance swum under the water surface. Placed next to the trunking, a plastic cone shows the 15 m and another the 20 m—only in view of the experimenter (and the swimmer’s when resurfaced). Each swimmer estimated the feasible distance they thought they could reach during apnea. Distances were restricted to 15 and 20 m. E15 and E20 refer to 15 and 20 m predictions, respectively. The start was underwater. When the swimmer resurfaced before 15 m, the game stopped, and the swimmer tried again without being penalized. When the swimmer exceeded 15 m and surfaced before 20 m, the trial was coded P15 (for “Perform 15 m”). A trial strictly or exceeding 20 m was coded P20 (for “Perform 20 m”). The following payoffs were known to everyone:

• E20-P20: the best estimation and performance, they scored four points.
• E15-P15: when the swimmer successfully performed their estimations, they scored three points.
• E20-P15: when the swimmer performed less than estimated they still pocketed two points, corresponding to a relative underperformance without compromising their security, an extremely important rule not to be neglected.
• E15-P20: when the swimmer performed more than estimated, they only scored + 1 point. This was the worst score because, in apnea, exceeding the estimated distances was potentially exceeding one’s limits (=danger).

**Matrix 1.** Payoffs associated with the two estimations (E15 and E20 for estimate 15 or 20 m) according to the performance swum (P15 and P20 for performance on 15 or 20 m).

**M1**	**P15**	**P20**

**E15**	**+3**	**+1**

**E20**	**+2**	**+4**

If swimmers were meaningful virtual players, they would first avoid the worst. The tactic of the lesser evil by playing E20, called “Maximin” (von Neumann and Morgenstern, 2007) allows scoring + 2 (i.e., by trying to swim as far as possible but unsuccessfully). By playing E15, a minimum profit could be obtained + 1. By playing E20, this minimum profit rose to +2 points (noted: Max (1, 2) = + 2). This choice was a reliable solution since it enabled the dominant strategy (E20, P20) = + 4 points: the optimal balance of the game. A bold estimate was, therefore, fostered. The “Minimax” tactic is defined by the minimum of the maximum satisfaction; it was given by Min (3, 4) = + 3. This Minimax also corresponded to a balance (E15, P15): resulting from a significant “comfort” strategy. This strategy was, however, less robust than (E20, P20). Nevertheless, greater consistency between estimations and realizations improved the scores (+3 and +4).

As swimmers had six trials in that game, the game did have a balance in mixed strategy (Nash, 1950): Let *p* be the probability of playing E15 and (1–*p*) the probability of playing E20. Then, the gain expectation if P15 is EP15 = 3 × *p* + 2 × (1–*p*) = *p* + 2 and the gain expectation if P20 was EP20 = 1 × *p* + 4 × (1–*p*) = 4–3*p*. To play the balance, we put EP15 = EP20, in other words, p + 2 = 4–3*p*; it came *p* = 1/2 and therefore EP15 = EP20 = 2.5 points. This result was better than Maximin but not as good as Minimax. It was assumed that *q* was the probability of P15 and (1–*q*) that of P20. Similarly, the equilibrium was in EE15 = EE20, i.e., 3 × *q* + 1 × (1–*q*) = 2 × *q* + 4 × (1–*q*). Then 2*q* + 1 = 4–2*q*, *q* = 3/4 (probability of playing P15—and therefore 1/4 the probability of P20—to obtain a score of 2.5 points). In other words, by deviating from the comfort zone: E15 with P15 (Minimax = + 3), the player loses only −0.5 points by playing every other E20 in the hope of succeeding (P20) only once in four times. Everything was done to give the swimmer confidence to take the risk over the longer distance (E20) without risk to the score (+2 if Maximin, +2.5 if the balance in mixed strategy, +4 if the balance in pure strategy: he, therefore, avoids the worst = + 1).

If the players acted like a well-programmed computer without problems of apnea or distance recognition, they would systematically play (E20, P20) to obtain the maximum score (+4). But what strategy will be implemented by swimmers? The caution of the Maximin (E20) with the possibility of a maximum score (when exceeding 20 m) counteracted the balance of the Minimax (E15). The Minimax appeared simpler in theory, but practically, this choice could have induced the worst score: + 1. The solution could have also been adopted by a mixed strategy (playing E15 half the time and based on the assumption of resurfacing in P15 three times out of four).

### Measures

The experimentation took place over the following three sessions with 1 week between each.

**FIGURE 1:**
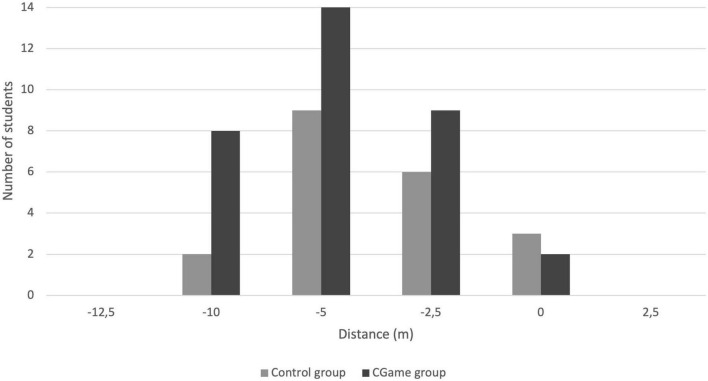
Difference of distance at pre-test apneas between estimation and the real distance performed for both groups. Vertically, the number of swimmers. Horizontally, the distance is in meters. The homogeneity test confirms that the groups can be considered as belonging to the same population (Chi^2^ = 2.78, dof = 1, *r* = 0.1, NS). They are only underestimated, most often 5 m below the estimated distance.

**FIGURE 2:**
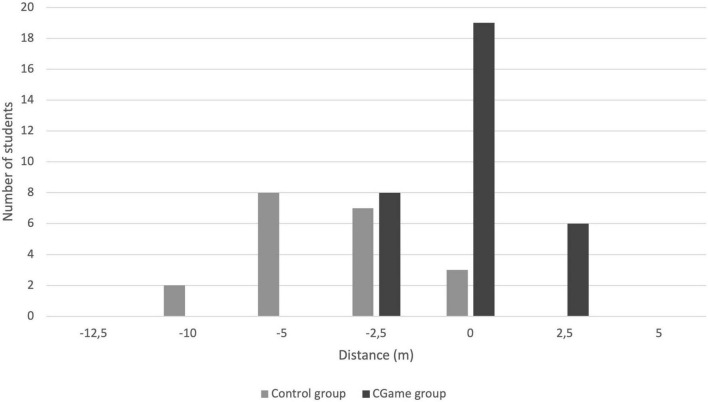
Difference of distance at post-test apneas between estimation and the real distance performed for both groups. The homogeneity test shows that the groups are to be considered significantly different (Chi^2^ = 21.99, dof = 1, *r* < 0.001). We notice that 19 of the 33-CGame group swimmers achieved a perfect match between saying and doing (concordance). The remaining 14 CGame participants only deviated by a maximum of 2.5 m from the estimation. For the first time, there was overestimating (for six swimmers of the CGame group).

(i) In the first session, an identical pre-test for selection is carried out for the 53 men. It simply consists of asking to “swim as far as possible underwater.” Starting in the water without equipment (except goggles), the swimmers go one by one. Distributed every 2.5 m on the poolside, pull buoys indicate distances from 5 to 25 m. Before starting their apnea, everyone must estimate the distance they will browse before resurfacing. There are no marks underwater. Then, once their maximum apnea has been achieved, the swimmers resurface and continue other crawl exercises (unrelated to the investigation) in the ancillary corridors. For each swimmer, the distance where he emerges as close as possible to the marks (pull buoys) is measured, making it possible to compare the predictions with the achievements (Figure 1). Swimmers have no feedback on their underwater performance and cannot trade right after. Exercise is perceived as part of their training of the day (these are sports students who prepare a performance in a 400-m crawl at the end of a cycle of 10 sessions: the pre-test is placed in the middle of this cycle and the middle of other exercises).

(ii) In the second session, the game was explained to 33 selected swimmers from the CGame group. Each swimmer had six trials to perform—interspersed with other swimming exercises (mainly crawling). To motivate participants, the 10 overall best players obtained at the end of the game a bonus point on their swimming score. Each student had to write the prediction on the board before each trial. When they finished, the distance performed was written just beside their estimation. Results of each “estimate” (E) and “performance” (P) were reported in Matrix 2 (M2). The total results (6 × 33 = 198 estimations followed by 198 dynamic apnea measurements), and the intermediate results at each stage of the game were presented, in order to see the possible strategic evolution (Matrix M3-M4-M5-M6-M7-M8). The payout expectation was calculated at each stage of the game. For their part, six maximum dynamic apneas (interspersed with other crawl situations) were offered to the non-recruited group of 20 swimmers. To provide feedback on performances when swimmers were resurfacing, pull buoys were placed on the side of the pool. No other action was taken with them regarding apneas.
(iii) The third session consisted of a post-test: the 53 sports students reiterated the pre-test (Figure 2). The results between estimations and achievements were compared between swimmers who participated in the CGame swimmer’s session (*n* = 33) and residual swimmers (*n* = 20).

## Results

Although their performance in maximum dynamic apnea was different, both the 33-CGame group and the 20 other students underestimated themselves during the first apnea (pre-test) in a way comparable to Chi^2^ with χ^2^ = 2.78, dof = 1, *r* = 1, difference not significant (Figure 1). A small proportion of swimmers managed to match prognosis and performance (5/53).

At the CGame itself, most swimmers first played Minimax = (E15, P15) = 49% (M2). Of the 198 passes, 36% played the optimum balance = (E20, P20), i.e., these players emerged to the surface at more than 20-m away, while none of the 33 swimmers selected reached this distance during the pre-test (Table 1). Concordant strategies are those whose estimated and performed swimming distances are the same. Conversely, discordant strategies are those whose estimated and performed swimming distances are different. During the game, concordant strategies were much more present than non-concordant strategies (E20, P15) and (E15, P20) which were observed in 13 and 2% of cases, respectively. In the end, the game itself yielded an average payout: EM2 = + 3.2 points per move, better than Minimax and Maximin, and less than the optimum balance in pure strategy (that a machine would have played).

**Matrix 2.** Distribution in the four strategies of Matrix 1 of the 198 trials.

**M2**	**P15**	**P20**

**E15**	**97 (49%)**	**5 (2%)**

**E20**	**25 (13%)**	**71 (36%)**

Strategies tended to evolve during the game. At the first step of the game (Matrix 3), the swimmers remained in their comfort zone [29 of the 33 players played (E15, P15)]. Only four of them—due to a lack of underwater landmarks—surfaced too far, beyond 20 m, and obtained the worst score = + 1. This fear of the worst gradually affected the whole group. Between Matrix 3 and Matrix 5, more than a dozen swimmers moved away from E15, and none were trapped in (E15, P20) = + 1. The expectation of gain increased significantly, from + 2.7 points in M3 to +2.9 points in M4 and M5. In M5, the swimmers’ way of playing was similar to the one given by Nash’s balance (*p* = 1/2 and *q* = 3/4, *E* = + 2.5).

**Matrices 3, 4, and 5.** Evolution of strategies during the first three trials of the game.

**M3**	P15	P20	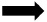	**M4**	P15	P20	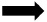	**M5**	P15	P20

E15	**29**	4		E15	**23**	1		E15	**18**	0

E20	0	0		E20	5	4		E20	9	6

In the middle of the game (Matrix 6), the distribution of strategies was more diverse than at the beginning (Matrix 3) and the end (Matrix 8). Henceforth, this distribution moved away from the probabilities rendered by the calculation (and observed in Matrix 5). In Matrix 6, it was rather *p* = 1/3 (11/33) and *q* = 1/2 (17/33). Symptomatic of greater self-confidence, Matrix 6 corresponds to the moment when more students chose the E20 tactic rather than the E15. The game switched at this time. The expectation of gain gains: EM6 = + 3.3: EM7 = + 3.5, EM8 = + 3.7. Players did better than the theoretical mixed balance gives. The improvement in the expectation of gain was also due to the gradual disappearance of divergent strategies [a single strategy: (E20, P15) in Matrix 8]. Comforted by a game mechanic that was benevolent toward risk (swimming over 20 m), most players ended up playing (E20, P20): 24 of the 33 swimmers in Matrix 8, whereas none of the 24 surfaced at 20 m during the first session.

**Matrices 6, 7, and 8.** Evolution of strategies during the last three trials of the game.

**M6**	P15	P20	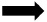	**M7**	P15	P20	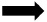	**M8**	P15	P20

E15	**11**	0		E15	**8**	0		E15	**8**	0

E20	6	**16**		E20	4	**21**		E20	1	**24**

**FIGURE 3:**
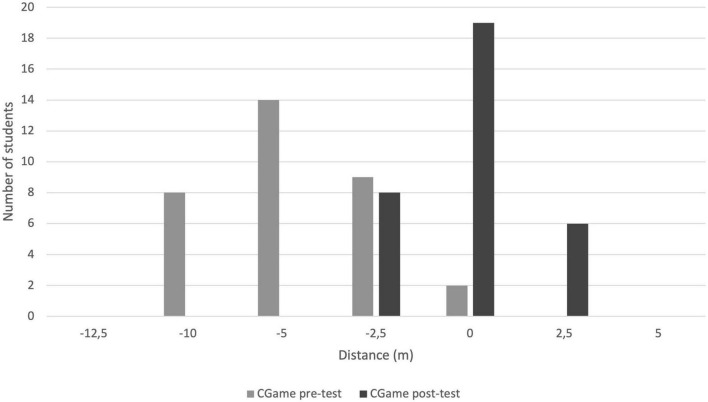
Difference between prediction and maximum pre-test and post-test apneas for the CGame group. The differences before and after the exercise of the CGame are significant (Chi^2^ = 41.82, dof = 1, *r* < 0.001). The conjunctions between saying and doing are much closer at the end of the game.

At the end of the game, during the post-test, the CGame group progressed in the estimation of the distances swum in apnea unlike the non-recruited group which stagnated (Figure 2). When there were differences, they were only underestimated for the CGame group (Figure 3). The CGame did influence the estimation of apneas.

## Discussion and conclusion

The present study reported significant progress in apnea performance in the CG group, but no evolution had been reported for the residual group. In addition, the best swimmers also improved their performance after six repetitions of maximum apneas. For each performance level, we could enlarge a suitable CGame, with E20 and E25 for the best and E10 and E15 for swimmers with fewer capabilities.

The most surprising result of this study is probably a lack of progress. The difference between estimated and performed apneas of the residual group is equivalent (except for one swimmer) before and after having done six maximum dives without instructions (Figure 1 *vs.* Figure 2). This lack of progress contrasts with the results obtained thanks to the implementation of the CGame (Figure 3). To match the predictions and achievements, all the CGame players reported adopting a strategy of counting the number of strokes in an attempt to match the predictions and achievements. For example, eight movements in breaststroke from the wall to 15 m followed by three additional ones to pass the 20 m. This objectification of underwater distances is very classic in sports swimming. Thus, Michael Phelps systematically made 10 undulations after each dive to reach the surface at the 15-m buoy (beyond which he would have been disqualified). In aquatic environments, visual cues are subordinate to kinesthetic information (Collard et al., 2007). It seems that in the absence of a challenge, control of movements by counting failed. Thus, at the post-test, no swimmers from the residual group declared to have counted their arm strokes.

The strategic evolution at the CGame for the 33 swimmers is quite close to rational behavior. The swimmers started by playing Minimax (+3). Then, some swimmers moved away from it by playing Nash’s balance in mixed strategy (*p* and *q* close to 1/2 and 3/4 in M5). They sometimes lost but never fell below + 2 (Maximin). The expectation of gain had not even stopped rising. Finally, almost all of them had oriented themselves toward the optimum balance in pure strategy (+4). The mechanics of the game have shaped behavior—like a prisoner’s dilemma (Tucker and Straffin, 1983) that invites admission of the crime, even if nothing has been committed, or a Dove vs. Hawk (Dawkins and Davis, 1976; Binmore, 1992) that induces the survival of conciliatory behaviors in the middle of the power struggle. The master of the game is not the master or the player but the game. As a result, the CGame seems to us to be an interesting pedagogical tool for those who want to provoke progress between estimates and realizations of apneas.

The objective of the CGame is to adjust the participants’ body scheme to the underwater space to ensure safety. Drownings are most often the result of a lack of awareness of their potential for action. Syncope is linked to a physiological characteristic of the human species (Silbernagl and Despopoulos, 2007). Hyperventilation—even if not forced—which usually prepares apnea, significantly reduces the level of carbon dioxide (CO_2_) in the blood but almost does not increase the level of oxygen (O_2_). However, the physiological mechanism that makes you want to breathe again at the surface (diaphragm spasm) is the level of blood CO_2_ which is too high. In fact, while the CO_2_ tolerance threshold has not yet been reached, the O_2_ level may be too low, and the body goes into standby. If the swimmer is alone at the pool, drowning is guaranteed. It is, therefore, essential to get to know your own dynamic apnea abilities, and to objectify the relationship to underwater space—by counting, for example, the number of strokes in dynamic immersion before going up. This is what the exercise of the CGame led to, without being imposed. Additionally, the experimental game theory is undoubtedly a promising tool to cross perceptions with motor skills.

One of the possible approaches of our study can be done by the 360° approach used (Lavega-Burgués et al., 2020; Martín-Martínez et al., 2021). Through an observational methodology and T-Patterns, we could find out differences pre–post test throughout the temporal distributions events (Pic et al., 2022).

We used a pedagogy aimed at improving students apnea swimming performance. Indeed, the objective was not only to teach values about safety in the water but also to help them improve their performance. To meet these needs, the use of motor praxeology introduced by Pierre Parlebas with the theory of sports games allowed the swimmers to evolve. Indeed, at the beginning of the cycle most of the students overestimated their ability to swim underwater (this is a problem in view of the danger of apnea, if the swimmer is unable to swim what he/she has estimated then the risk of drowning is increased), this tendency was reversed at the end of the cycle. In parallel with the improvement in the estimation of their underwater swimming ability, swimmers saw their underwater swimming distance increase. The “Concordance Game” could be offered as a pedagogical tool in Physical Education or a sports club to learn to swim a longer distance below the surface without forcing and with respect for the safety rules.

To conclude, in view of the results obtained from “The Concordance Game,” we can say that it is an interesting pedagogical tool. It allowed the students to improve their dynamic apnea performance, by knowing more about their limits and by avoiding working in an unsafe way.

## Data availability statement

The raw data supporting the conclusions of this article will be made available by the authors, without undue reservation.

## Ethics statement

Ethical review and approval was not required for the study on human participants in accordance with the local legislation and institutional requirements. The patients/participants provided their written informed consent to participate in this study.

## Author contributions

LC designed and directed the project and performed the experiments. DV directed the project and developed the theoretical framework. DH wrote and translated the article. All authors thought about the method and discussed the results.
